# Simultaneous effect of naringenin and beta-catenin signaling inhibitor C-82 on modulating gene expression and functional pattern of mesenchymal stem cells from endometriosis patients

**DOI:** 10.22038/ijbms.2025.80388.17401

**Published:** 2025

**Authors:** Hoda Fazaeli, Faezeh Davoodi, Azar Sheikholeslami, Mohsen Sheykhhasan, Naser Kalhor, Leyla Naserpour, Rahil Jannatifar, Seyedeh Saeideh Sahraei

**Affiliations:** 1 Department of Cell Biology & Regenerative Medicine, Academic Center for Education, Culture, and Research (ACECR), Qom Branch, Qom, Iran; 2 Department of Reproductive Biology, Academic Center for Education, Culture, and Research (ACECR), Qom Branch, Qom, Iran

**Keywords:** Cell proliferation, Endometriosis, Inflammation, Mesenchymal stem cells, Survival

## Abstract

**Objective(s):**

One of the leading causes of endometriosis is the return of menstrual blood flow into the pelvic cavity and the establishment of menstrual blood mesenchymal stem cells (MenSCs) outside the uterus. MenSCs from endometriosis patients (E-MenSCs) and healthy women have been shown to vary in terms of surface markers and gene expression, which may suggest the involvement of these cells in the development and expansion of ectopic lesions. This study aimed to investigate the effects of beta-catenin signaling inhibitor C-82 and naringenin as PI3K signaling pathway inhibitors on E-MenSCs to modulate their gene expression and functional pattern.

**Materials and Methods:**

Briefly, E-MenSCs isolated by density-gradient centrifugation were treated with C-82 and naringenin, and the genes and pathways related to inflammation, proliferation, and survival were evaluated. E-MenSCs showed increased early apoptosis and decreased levels of ROS, IL-6 and IL-8, ER, α-SMA, and Ki-67 protein expression.

**Results:**

Our results shed light on the function of C-82 and naringenin in modulating E-MenSCs.

**Conclusion:**

However, more research is needed to analyze the precise effects of small molecule C-82 and naringenin on endometriosis.

## Introduction

Endometriosis (EM), as a common disease in almost 35–50% of women of reproductive age, causes severe pelvic discomfort and infertility (1, 2). The etiology of the disease is almost unclear yet, and the therapy is focused on surgery and hormone treatment using gonadotropin-releasing hormone (GnRH) agonists, progestins, and androgens (3, 4). However, hormone therapies for EM cause side effects, whereas the incidence of recurrence is about 50–60% after hormone therapy (5). Due to the limitations of therapeutic approaches for treating EM, studies aimed to clarify the underlying mechanisms could potentially provide a novel strategy for more effective treatment of deep EM.

On the other hand, the central hypothesis for the initiation of EM is retrograde menstruation and implantation of endometrial cells into the peritoneum cavity, leading to a disrupted concentration of inflammatory cytokines related to circulating concentrations of estrogens (6). According to the implantation theory, cell migration and fibrosis are critical processes for EM establishment (7, 8). Evidence of studies revealed that menstrual blood-derived mesenchymal stem cells (MenSCs) show different expression of genes and proteins of major signaling pathways in EM-affected women compared to healthy women, which may lead to retrograde menstruation and EM formation (9-11). Studies have shown that improper activation of the Wnt/β-catenin and the phosphatidylinositol-4,5-bisphosphate 3-kinase (PI3K)/AKT signaling pathways can play an important role in the migration and invasion of endometrial cells in patients with EM (4, 12-14). 

It has been reported that endometrium and endometriotic tissues from EM patients show an abnormal activation of the Wnt/β-catenin signaling pathway during the mid-secretory phase (15). β-catenin is crucial in regulating the cell cycle, which includes proliferation, differentiation, and migration in ectopic lesions (16). The liver, lung, kidney, and skin all have fibrotic disorders triggered by the Wnt/β-catenin signaling pathway. There are some reports about the relationship between fibrosis and the progression of EM (17); therefore, targeting fibrosis can be a new treatment strategy for EM.

ICG-001 and C-82 are two metabolites of PRI-724, a drug under clinical trials for its antitumor activity. These components are cyclic AMP response-element binding protein-binding protein (CBP)/β-catenin inhibitors (4, 13, 18). Through Wnt/catenin signaling, ICG-001 and C-82 prevented fibrosis (19). Furthermore, ICG 001 has shown excellent efficiency in increasing apoptosis and preventing migration, and C-82 demonstrated significant suppression of proliferation and cell viability (19). 

On the other hand, some interesting data show a potential involvement of the PI3K/AKT signaling pathway in the pathophysiology of EM (20, 21). Endometriotic stromal cells have been shown in studies to be unable to control PI3K/survivin signaling and cell growth in ectopic endometriotic epithelial cells (22). Increased activity of PI3K-AKT pathways was associated with nuclear factor-κB (NFκB) signaling in endometriotic cells (23). Studies showed that inhibiting the PI3K-Akt pathway improved endometriotic cells’ inflammatory and apoptotic conditions (24, 25).

Naringenin is a member of the family of flavanones, which are well-known for suppressing proinflammatory cytokines and having antiproliferative and anti-oxidant activities (26, 27). Naringenin also modifies estrogen receptor (ER) alpha signaling by interfering with the activation of extracellular signal-regulated kinases (ERK1/2), mitogen-activated protein kinase (MAPK), and PI3K signaling pathways (1, 28). Therefore, patients with EM may benefit from the therapeutic use of naringenin (1). 

Considering the importance of Wnt/β-catenin and PI3K-Akt signaling pathways in the development of endometriosis, and based on our previous studies declaring the differential gene expression pattern of NE-MenSCs and E-MenSCs (10, 29), we aimed to evaluate the inhibitory effects of naringenin (an AKT inhibitor), and C-82 (a β-catenin inhibitor), as well as their simultaneous effects on improving the MenSCs from EM patients.

## Materials and Methods

### Patient selection

This experimental study was conducted on menstrual blood samples from women referred to the Rooya Infertility Treatment Center, Qom, Iran. The ethical code was obtained from the Islamic Azad University Ethics Committee (IR.IAU.QOM.REC.1400.026), and all participants signed written informed consent. In the experimental group, women with endometriosis in stages III or IV of the disease were identified by a gynecologist through laparoscopy. According to the revised classification of the American Fertility Society, they were selected (E-MenSCs, n=5). EM cases involved women aged 27 to 35 who had irregular menstrual cycles with pelvic pain or suspected pelvic masses and who had not received hormonal therapy or surgery for at least three months.

### Isolation and characterization of MenSCs

The menstrual blood samples were obtained from EM patients through days 2–3 of the menstruation cycle. Based on the density gradient method, stromal cells were isolated from the samples using Ficoll-Paque (Lymphodex, Innotrain, Germany). They were cultivated in DMEM (Gibco, Grand Island, USA) with 10% FBS (Gibco, Grand Island, USA) and 1% penicillin/streptomycin (Gibco, Grand Island, USA) and incubated at 37 °C, 5% CO_2_, and 95% humidity. CD44, CD73, CD90, and CD105 were selected as positive markers, while CD34, CD45, and CD38 were analyzed as negative markers to confirm isolated MenSCs. PE-conjugated monoclonal antibodies against CD34, CD38, and CD73, as well as FITC-conjugated monoclonal antibodies for CD44, CD45, CD90, and CD105, were purchased from BD Biosciences (San Jose, CA, USA). Flow cytometry using an FC500 flow cytometer (Beckman Coulter, Fullerton, CA, USA) was performed, and the results were analyzed using Beckman Coulter software.

### Preparation of materials and study design

The study was designed in 4 groups, which were defined as follows:

1. Control group (C): MenSCs from endometriosis patients (E-MenSCs) with no treatment

2. Small molecule group (SM): E-MenSCs treated with C-82 

3. Naringenin group (Nr): E-MenSCs treated with naringenin 

4. Small molecule and naringenin group (SM/Nr): E-MenSCs treated with C-82 and naringenin

After reaching 70% confluence in passage 3, the cells were seeded in proper dishes for 24 hr. Then, the small molecule group and the naringenin group were cultured with fresh medium containing 2 μM small molecule C-82 (Sigma-Aldrich, Lyon, France) (30) and 100 μM naringenin (Sigma-Aldrich) (28), respectively. The exact amount of C-82 and naringenin for SM and Nr groups were used simultaneously for treating the SM/Nr group. The control group received no treatment. The time of treatment for all groups was 72 hr.

### Annexin V/propidium iodide (PI) staining

After 72 hr, the apoptosis was analyzed using an Annexin V/PI detection kit (BD Biosciences, Franklin Lakes, NJ, USA). Briefly, the cells were cultivated in 6-well plates to be treated and incubated for 72 hr at 37 °C in a CO_2_ incubator. Then, Annexin V-FITC antibody conjugated with PI was added to the prepared binding buffer for 30 min in the dark at 4 °C. Then, the cells were subjected to flow cytometry at the emission wavelength of 488 nm (FACS Calibur BD Biosciences).

### ELISA assay for quantification of secreted factors

The cells were seeded in 6-well plates (3×10^5^ cells/well). The expression level of Cas3, Cas7, interleukin (IL)-6, IL-8, and ROS was detected using an ELISA kit (R&D Systems) according to the manufacturer’s protocol. Briefly, conjugated antibodies were added to each well and incubated for 2 hr at room temperature. After washing the wells, substrate solution was added for 20 min in the dark, and then the stop solution was administered. The optical density (O.D.) for each sample was measured in 450 nm wavelength, and a standard curve was drawn based on the concentration of the standards. The concentration of markers in each well was calculated based on the slope of the line. The student’s t-test was used to calculate the statistical difference. *P*-value of *P*≤0.05 was considered to be statistically significant.

### Western blotting for quantification of secreted factors

Total protein was extracted from MenSCs of each group, and sodium dodecyl sulfate-polyacrylamide gel electrophoresis (SDS-PAGE) was used for protein separation based on molecular weight. After electrophoresis, the proteins were transferred to PVDF membranes. The membranes were immediately treated in a blocking buffer containing 5% BSA and 5% milk (Sigma-Aldrich, St. Louis, USA) for one hour at room temperature. Antibodies diluted in a blocking solution were used to probe the membranes at 4 °C overnight. The membranes were rinsed thrice for five minutes the following day in Tris-buffered saline containing 0.1% Tween® 20 (TBST). Then, the membranes were exposed to horseradish peroxidase-conjugated secondary antibodies in a blocking buffer for one hour at room temperature. After washing with TBST buffer, the blots were probed with antibodies against α-SMA, β-catenin, ER, Ki67, and β-actin. An image analyzer called LAS 3000 (GE Healthcare, Little Chalfont, UK) was used to digitize blot images. Using ImageQuant 5.2 software, band intensities were analyzed (GE Healthcare, Little Chalfont, UK).

### Real-time PCR analysis

The purity and quantity of total RNA extracted using the RNeasy kit (Gene All Biotechnology, Seoul, Korea) were analyzed with a Nanodrop 2000 spectrophotometer (Thermo Fisher Scientific, Wilmington, USA). Then, cDNA was synthesized using a reverse transcription Kit (HyperScript™ RT-PCR Premix, GeneAll Biotechnology, Korea). After that, RealQ Plus Master Mix (AMPLIQONIII) was used to quantify the mRNA expression level of BAX, BCL2, COL1, FOXP, α-SMA, and β-catenin genes (Table 1) based on the manufacturer’s instructions. Briefly, a mixture was made containing 5 μl of Master Mix, 1 μl of cDNA, and 1 μl of forward and reverse primers. A final volume of 10 μl was achieved by adding millipore water. Initial denaturation at 95 °C for 10 min was followed by 40 cycles of 10 sec denaturation (95 °C), 40 sec annealing (58 °C), and 35 sec extension (72 °C). The experiments were done three times to quantify target genes. Also, GAPDH was used as a housekeeping reference to normalize the gene expression levels.

### Statistical analysis

SPSS 26 (SPSS, Chicago, IL, USA) was used to evaluate the results. The statistical analysis was done using one-way ANOVA, Tukey *post hoc* test, and paired-samples T-test. The *P*-value<0.05 was considered significant, and the data were presented as mean±SD.

## Results

### Characterization of E-MenSCs

**Figure 1:**
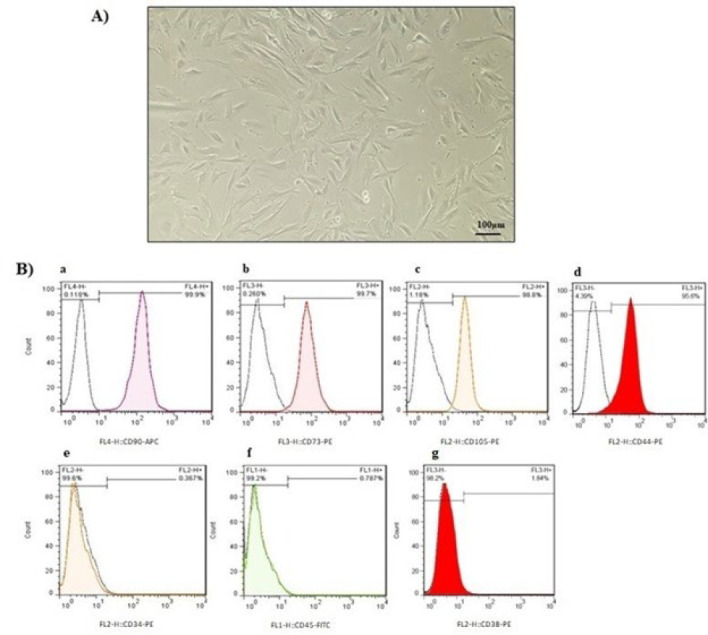
Typical characteristics of E-MenSCs

During primary culture, MenSCs exhibited radial or helical growth patterns and spindle-shaped, fibroblast-like morphologies typical of MenSCs (Figure 1A). Additionally, based on the obtained flow cytometric data, E-MenSCs expressed CD44, CD90, CD73, and CD105 as MSC surface markers and were negative for CD34, CD38, and CD45 as hematopoietic stem cell markers (Figure 1B). 

### Apoptosis assessment

*Annexin V/PI assay*

**Figure 2:**
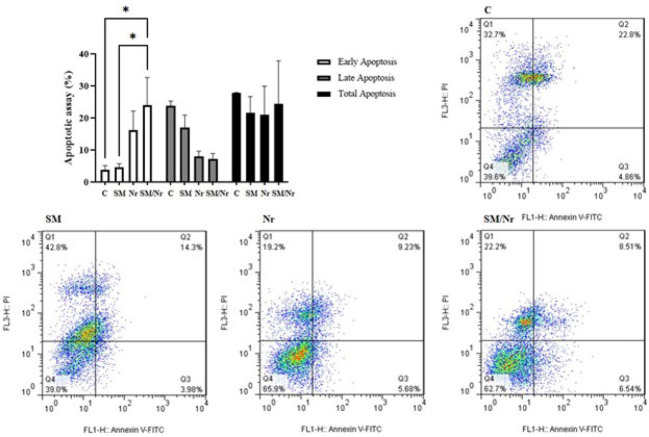
Flowcytometric apoptosis evaluation using Annexin V/Propidium Iodide assay in untreated (C) and treated E-MenSCs (SM, Nr, and SM/Nr)

The effects of applied treatments (C-82 and naringenin, alone or together) on the apoptosis of E-MenSCs were assessed through flow cytometry with annexin V and PI detection kit. As expected, the obtained data showed a significant increase in early apoptosis of combination treatment of naringenin with C-82 in comparison with untreated E-MenSCs (P=0.02) and C-82 group (P=0.02), while although C-82 and naringenin alone increased the early apoptosis percentage in treated cells. However, they did not elevate it in a statistically significant way (P=0.99 and P=0.21, respectively). However, it was demonstrated that none of the applying treatments could exert a significant change in the percentage of late and total apoptosis (P>0.05) (Figure 2).

*BAX/BCL2 gene expression*

**Figure 3:**
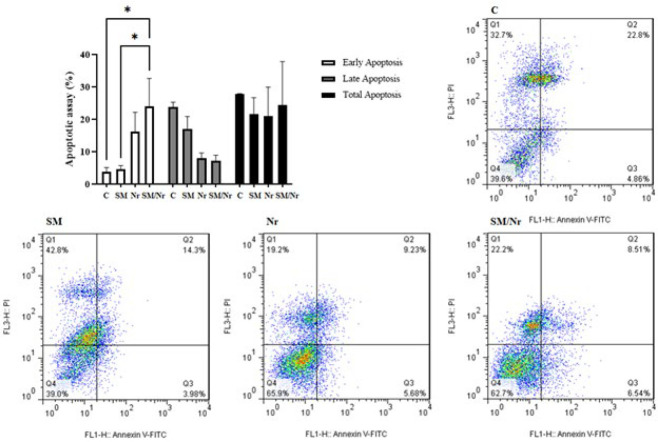
Comparison of the BAX, BCL2, and BAX/BCL2 ratio in C, SM, Nr, and SM/Nr groups using Real-Time PCR technique

Next, we evaluated genes related to apoptosis. Our findings revealed that both C-82 and the combination of C-82 and naringenin significantly increased the expression of BAX (a pro-apoptotic gene) and BCL-2 (an anti-apoptotic gene) compared to the control group (*P*=0.00). In contrast, no significant changes were observed in the Nr group (*P*>0.05). So, when the BAX/ BCL2 ratio was assessed, none of the treated groups were different from the untreated E-MenSCs (*P*>0.05) (Figure 3). 

*Caspase 3/7 activity*

**Figure 4:**
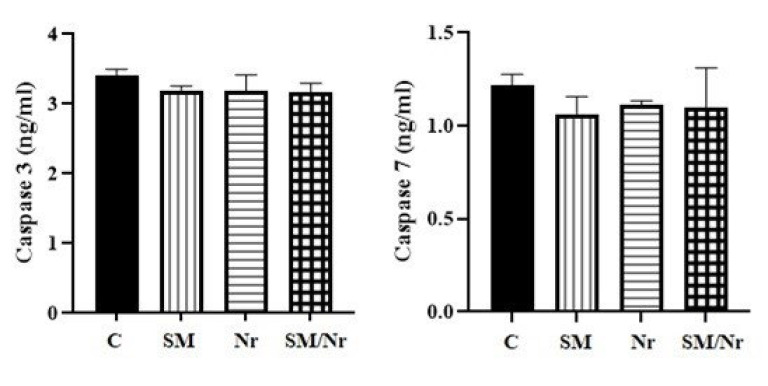
Comparison of Caspase 3 and 7 in different groups of E-MenSCs (C, SM, Nr, and SM/Nr) using ELISA assay

We showed that none of the applying treatments significantly changed the late and total apoptosis percentage in the EM cell line. These results were confirmed in analyses for caspase 3/7 activity based on the ELISA analysis data. As expected, no significant difference in caspase 3/7 activity of endometrial stromal cells was observed between the treated groups and the control group (*P*>0.05) (Figure 4). Treatment with C-82 and naringenin did not cause a significant change in caspase 3/7 activity.

*The level of ROS*

**Figure 5:**
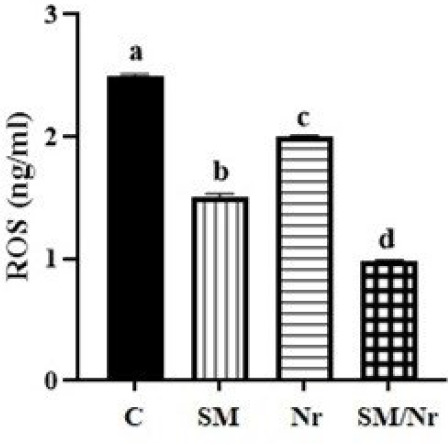
Evaluation of ROS concentration in different groups of E-MenSCs (C, SM, Nr, and SM/Nr) using ELISA assay

Surprisingly, naringenin and C-82 treatment could decrease the level of ROS production in the EM cell line (*P*=0.0001), with a priority for combination treatment of C-82 with naringenin compared to C-82 and naringenin alone (*P*<0.0001) (Figure 5).

*The levels of IL-6 and IL-8*

**Figure 6:**
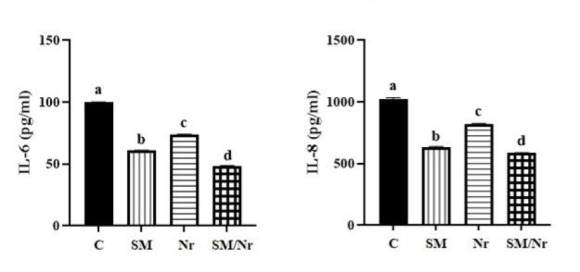
Evaluation of IL-6 and IL-8 concentration in different groups of E-MenSCs (C, SM, Nr, and SM/Nr) using ELISA assay

The levels of inflammatory factors, including IL-6 and IL-8, significantly decreased in all C-82, naringenin, and combined C-82/naringenin groups compared with untreated E-MenSCs (*P*<0.0001). Moreover, when we compared treated groups, it was shown that combination treatment with C-82 and naringenin was more efficient in decreasing both IL-6 and IL-8 in E-MenSCs (*P*<0.0001) (Figure 6).

*The expression level of β-catenin*

**Figure 7:**
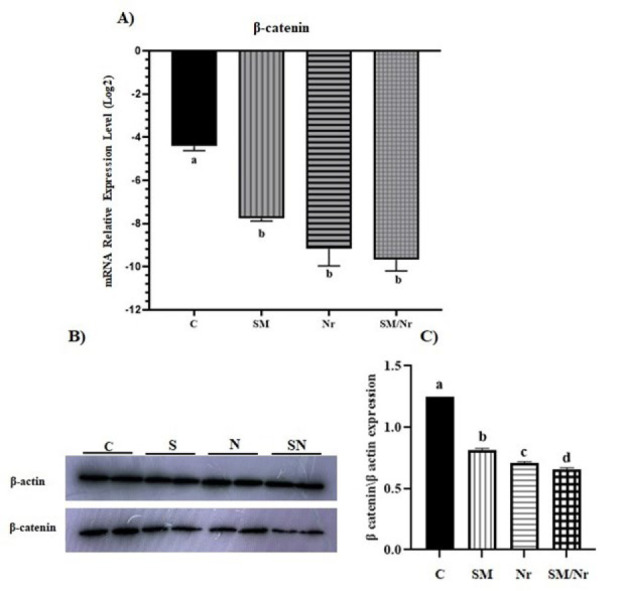
Effect of different treats including C-82 (SM), Naringenin (Nr), and C-82 and Naringenin (SM/Nr) on the levels of β-catenin in the E-MenSCs

As shown in Figure 7a, the expression level of the β-catenin gene showed a significant decrease in all treated groups compared to the untreated E-MenSCs (*P*=0.05). Also, the protein expression level of β-catenin showed a significant decrease in all the treated groups compared to the control group. This reduction was more significant when C-82 was combined with the naringenin treatment group (*P*=0.0001) (Figure 7 b, c).

*The expression level of* *α* *-* *SMA*

**Figure 8:**
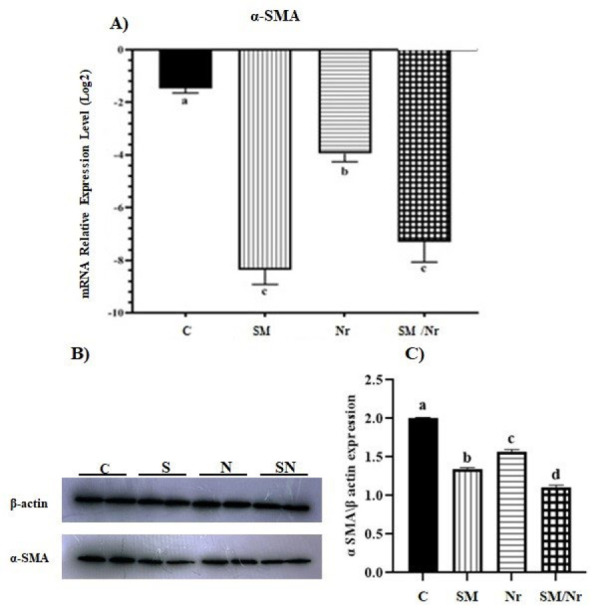
Effect of different treats including C-82 (SM), Naringenin (Nr), and C-82 and Naringenin (SM/Nr) on the levels of α-SMA in the E-MenSCs

In the case of the α-SAM (alpha-smooth muscle actin, a fibrotic marker) gene, significant down-regulation was detected in all C-82, naringenin, and combined C-82/naringenin treated groups (*P*=0.0001, *P*=0.006, and *P*=0.0001, respectively) (Figure 8a). Moreover, this down-regulation in both C-82 and combination C-82 with naringenin groups was more efficient than in the Nr group compared to untreated E-MenSCs (*P*=0.0001 and *P*=0.001, respectively). Also, the protein expression level of α-SMA was examined by western blot analysis. As expected, α-SMA protein significantly decreased in all treated groups (*P*=0.0001). Moreover, the combination treatment with C-82 and naringenin showed a more efficient reduction than the control group (*P*=0.0001) (Figure 8 b, c).

*The expression of estrogen receptor*

**Figure 9:**
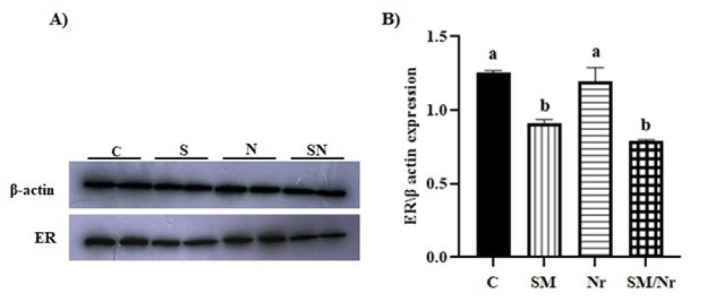
Evaluating ER concentration in different groups of E-MenSCs (C, SM, Nr, and SM/Nr) using western blot

Compared to the control group, ER (Estrogen Receptor) protein showed a significantly decreased expression level in both C-82/naringenin and C-82 groups (*P*=0.0001 and *P*=0.0001, respectively). In contrast, treatment with naringenin did not result in decreased expression (*P*=0.434). It is worth mentioning that there was no significant difference between the ER expression level of C-82 and combined C-82 with naringenin groups (*P*=0.0555) (Figure 9).

*The expression level of Ki67*

**Figure 10:**
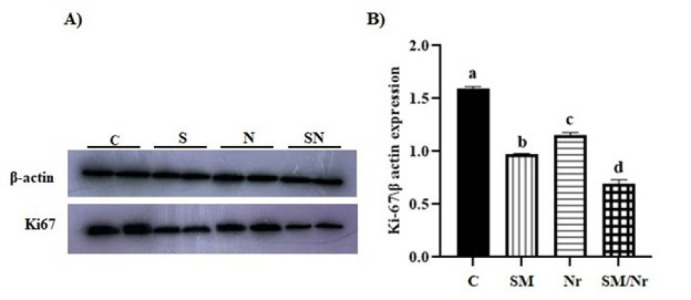
Evaluation of Ki-67 concentration in different groups of E-MenSCs (C, SM, Nr, and SM/Nr) using western blot

Western blot analysis revealed that the expression level of Ki67 was decreased in all treatment groups. However, the expression level of Ki67 was decreased more efficiently in the C-82/naringenin group as compared with other treated groups (*P*<0.0001) (Figure 10).

*Fibrotic marker gene expression*

**Figure 11:**
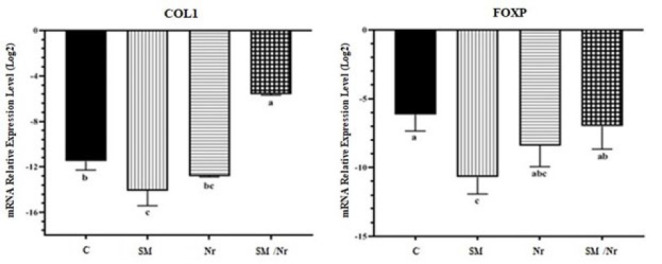
Comparison of the COL1 and FOXP genes in C, SM, Nr, and SM/Nr groups using Real-Time PCR technique

COL-1 and FOXP1 are fibrotic genes that are involved in fibrosis during EM. The mRNA level of COL-1 in C-82 treatment was down-regulated in comparison to the control group (*P*=0.004) whereas, Col-I expression was up-regulated in combination with C-82 with naringenin treatment in comparison to the control group (*P*=0.000) (Figure 11). In the case of FOXP1 expression, after receiving treatments, only the C-82 group experienced significant down-regulation (*P*=0.002). COL-1 and FOXP1 expression in E-MenSCs was not affected by naringenin treatment.

## Discussion

Studies have demonstrated that the migration and invasion of endometrial cells in the menstrual blood of patients with EM can be significantly influenced by the abnormal activation of the Wnt/β-catenin and the PI3K/AKT signaling pathways (10, 31). Furthermore, there is evidence that estrogen and progesterone can modulate EM by controlling the Wnt/β-catenin and PI3K/AKT pathway (10). Given that the herbal medication naringenin can inhibit the PI3K/AKT pathway, as well as the ability of small molecule C-82 to inhibit the Wnt/β-catenin pathway, this work used to investigate the simultaneous inhibition of these two pathways in endometrial stem cells derived from the menstrual blood of EM patients. Their effects have been discussed by examining the ability of proliferation, survival, and differentiation. 

In the current study, naringenin enhanced the level of the early apoptosis pathway, similar to the results of previous research. On the other hand, evidence points to the anti-inflammatory and tolerogenic effects of early apoptotic cell absorption (32, 33). Naringenin is used to treat EM cells because EM cells have high levels of inflammation and low levels of apoptosis (32, 33). This causes both apoptotic activity and anti-inflammatory activity to be triggered, which lowers inflammation after early apoptosis (32, 33). 

According to research, naringenin’s pro-apoptotic action is mediated by mitochondrial dysfunctions and caspase activation, which are related to the inactivation of the PI3K/Akt pathway in a lymphoma cell line (1). The activation of nuclear factor kappa (NF-κB)-light-chain-enhancer of activated B cells, may be one of the methods through which naringenin induces apoptosis (34). In the Zuo *et al*. study, the process of naringenin-induced K562 apoptosis was markedly enhanced by the enzyme activity of caspase-3 and caspase-8 (35). In the current study, treating small molecule C-82 and naringenin did not affect caspase 3/7 activity. Furthermore, in agreement with previous studies, naringenin resulted in a higher increase in early apoptosis than the small molecule C-82 group alone. In addition, the treatment of small molecule C-82 along with naringenin promoted early apoptosis compared with the control group but did not affect late and total apoptosis. 

Evidence showed that endometriotic cells had greater levels of endogenous oxidative stress, as demonstrated by an increase in the formation of ROS, changes in the ROS detoxification pathways, and a decrease in the catalase levels (36). An experimental study reported that naringenin’s anti-oxidant capacity has been thoroughly investigated in several cell lines and animal models (37). Scavenging free radicals and blocking the actions of pro-oxidant enzymes such as nicotinamide adenine dinucleotide phosphate (NADPH) oxidase, cyclooxygenase, lipoxygenase, and xanthine oxidase, and metal ion chelation are some of the naringenin’s best-studied effects (37). It was observed that naringenin increases the levels of several anti-oxidant enzymes, including catalase, glutathione peroxidase, and superoxide dismutase; inhibits protein nitration; and prevents peroxynitrite-induced protein oxidation (38, 39). According to the findings of a study, elevated ROS were inhibited both *in vitro *and in a mouse model of EM by the small molecule N-acetyl-cysteine (36). Our findings support those of earlier research. So, the results of the present study showed that ROS levels were remarkably decreased in treatment groups (C-82, naringenin, and C-82 along with naringenin groups) compared to the EM groups. Furthermore, the ROS level in the small molecule C-82 group was observed to be decreased compared to the naringenin group. Moreover, the combination of two inhibitors (C-82 and  naringenin groups) has reduced the level of ROS better than the C-82 and naringenin groups alone, so it has given better results regarding the effect on the simultaneous inhibition of two pathways.

Germeyer *et al*. observed that using metformin small molecules could regulate IL-8, IL-1β, ICAM, and IGFBP-1 expression in human endometrial stromal cells (40). In their study, IL-8, IL-1β, ICAM, and IGFBP-1 were less expressed in the treatment group than in the control group (40). Similarly, the present study showed that IL-8 protein expression in the naringenin group was lower than in the small molecule C-82 group. Many research studies reported that IL-6 is a key proinflammatory molecule that influences the onset and progression of EM via the cytokine network. In addition, through interfering with cellular immunological function, IL-6 also plays a significant role in the pathogenesis of EM (41). Furthermore, according to evidence, IL-6 levels in the peritoneal fluid are higher in EM patients. Furthermore, the results of the present study demonstrated that IL-6 protein expression in the combination of small molecules (C-82 along with naringenin groups) displayed a decreased level compared to the small molecule C-82 group.

Evidence suggests that aberrant activation of the Wnt/β-catenin pathway may contribute to the pathophysiology of EM (42). ICG-001, as a β-catenin inhibitor, decreased the growth of endometriotic lesions in the animal model (13). Our investigation found that the β-catenin gene and protein expression were remarkably decreased in treatment groups compared to the control group. The administration of small molecule C-82 and naringenin decreased the β-catenin expression compared to the control group and led to a more significant decrease than the C-82 and naringenin groups alone. Furthermore, the results of the present study demonstrated that β-catenin expression in the Nr group displayed a decreased level compared to the SM group. Although the Wnt/β-catenin and PI3K/AKT signaling pathways were investigated separately and showed promising results, our work revealed that combined suppression of these two signaling pathways, which are involved in the development of EM, can have a more significant impact. 

Smooth muscle hyperplasia and hypertrophy in endometriotic lesions exhibit consistent expression of α-SMA (43). In addition, the expression of α-SMA, smooth muscle density, and degree of pain in EM were all strongly associated (43). It was found that treatment with C-82 significantly decreased the expression of α-SMA mRNA in the stromal cells of endometriotic cysts (ECSCs) (30). Western blot showed that ECSCs had much higher protein expression of α-SMA than normal endometrial stromal cells, although neither C-82 nor ICG-001 significantly changed this expression in ECSCs (13). Our results are in line with those that have been reported in the vast majority of earlier examinations. Our findings showed that α-SMA expression was remarkably decreased in treatment groups (SM, Nr, and SM/Nr groups) compared to the control groups. Furthermore, the present study’s findings showed that α-SMA expression in the small molecule C-82 group was lower than in the naringenin group.

According to another research (2020), COL-I levels were shown to be higher in tissues with severe EM than in those with modest fibrotic alterations or healthy endometria (44). It was also observed that COL-I gene expression may be increased in EM (45). In addition, the findings of the present investigation showed that COL-I gene expression was lower in the SM/Nr group than in the other groups. 

In EM, FOXP1 increases fibrosis by triggering the Wnt/β-catenin signaling pathway. The current study proved that C-82 and naringenin treatment induced FOXP1 gene expression more significantly than the SM group alone and compared to the Nr group alone. 

A study revealed that EM patients have abnormal endometrial stromal cell function and increased endometrial ER expression during the secretory phase (46). The results of the study conducted by Shebley *et al*. showed that the small molecule elagolix could regulate circulating estrogen levels in EM patients, suppressing gonadotropin hormones and ovarian estrogen biosynthesis in a dose-dependent manner, reducing estradiol (E2) to a virtually full extent at higher dosages (47). According to a study’s results, naringenin influences estrogen receptor alpha signaling as well as the ERK1/2, MAPK, and phosphatidylinositol-4, 5-bisphosphate 3-kinase (PI3K) signaling pathways (28). Following earlier research findings, our study’s results showed that the ER protein expression was markedly lower in the SM/Nr group than in the control group. Furthermore, the results of the present study demonstrated that ER protein expression in the SM group displayed a decreased level compared to the Nr group. The SM was able to change estrogen level. However, Nr has not changed, so one of the advantages of simultaneously inhibiting two pathways can be that at least one important signaling pathway is inhibited.

In a study conducted by Park et al., the Ki-67 proliferation index was used to measure endometrial cell proliferation in patients with EM, and it was discovered that this proliferation was higher than that in individuals without EM (48). Ki67 expression is frequently employed as a cell proliferation marker and is easily observable via immunocytochemistry (49). In the study performed by Luo et al., ectopic lesions showed a greater incidence of Ki-67 expression than eutopic endometrium (50). Another study discovered that the administration of phytoestrogen-containing soy extract (SSE) significantly decreased the epithelium expression of the proliferative marker Ki67 in comparison to SHAM and ovari-ectomized (OVX) rats (51). These findings were consistent with our study results (52). Our results demonstrated that the treatment groups’ expression of the Ki-67 protein was substantially lower than that of the control group. Addi-tionally, the present study’s findings showed that the expression of the Ki-67 protein was lower in the Nr group than in the SM group. Our research demonstrated that there is a better outcome when these two signaling pathways that contribute to the development of EM are inhibited together.

The main limitation of our study was that, although previous research has explored the mechanisms of action of these two small molecules, our focus was on assessing the inhibitory effects of naringenin (an AKT inhibitor) and C-82 (a β-catenin inhibitor). Additionally, we aimed to evaluate the combined impact of these compounds on improving MenSCs from EM patients. Therefore, it would be beneficial to investigate the direct mechanisms of action of these small molecules while also examining the proteins that function downstream in these pathways.

**Table 1:** specific primers of human target genes for Real-time PCR

Product size	Accession number	Sequence	Gene
149	NM_001291430.2	F: CGGCAACTTCAACTGGGG R: TCCAGCCCAACAGCCG	BAX
114	NM_000657.3	F: GGTGCCGGTTCAGGTACTCA R: TTGTGGCCTTCTTTGAGTTCG	BCL-2
119	NM_001141945.2	F: GTGTGACAATGGCTCTGGG R: GTCCCATTCCCACCATCAC	α-SMA
146	NM_000088.4	F: GAAGACATCCCACCAATCAC R: CAGTTCTTGGTCTCGTCACA	COL1
203	NM_001330729.2	F:GCGTGGACAATGGCTACTC R:GCCGCTTTTCTGTCTGGTT	β-catenin
104	NM_001349338.3	F: ATGAACGGATGGATGTGATG R: ATAAAAAGCCTGGGGTCACT	FOXP

(A) Morphology of E-MenSCs. (B) The expression of E-MenSC surface markers was detected by flow cytometry so that they were positive for CD90, CD73, CD105, and CD44 (a-d) while negative for hematopoetic surface markers CD34, CD45, and CD38 (e-g). E-MenSCs: MenSCs from endometriosis patients.

Total apoptosis has not changed significantly in any of the treated E-MenSCs groups. Q1: Necrosis Q2: Late Apoptosis Q3: Early Apoptosis Q4: Live Cells. E-MenSCs: MenSCs from endometriosis patients; SM: small molecoule (E-MenSCs treated with C-82); Nr: Naringenin (E-MenSCs treated with naringenin); SM/Nr: E-MenSCs treated with C-82 and naringenin

Bars in different letters represent significant difference. C; MenSCs from endometriosis patients (E-MenSCs), SM; small molecoule (E-MenSCs treated with C-82); Nr: Naringenin (E-MenSCs treated with naringenin); SM/Nr: E-MenSCs treated with C-82 and naringenin

Bars in different letters represent significant difference. C; MenSCs from endometriosis patients (E-MenSCs), SM; small molecoule (E-MenSCs treated with C-82); Nr: Naringenin (E-MenSCs treated with naringenin); SM/Nr: E-MenSCs treated with C-82 and naringenin

Bars in different letters represent significant difference. C; MenSCs from endometriosis patients (E-MenSCs), SM; small molecoule (E-MenSCs treated with C-82); Nr: Naringenin (E-MenSCs treated with naringenin); SM/Nr: E-MenSCs treated with C-82 and naringenin

Bars in different letters represent significant difference. C; MenSCs from endometriosis patients (E-MenSCs), SM; small molecoule (E-MenSCs treated with C-82); Nr: Naringenin (E-MenSCs treated with naringenin); SM/Nr: E-MenSCs treated with C-82 and naringenin

A) gene expression level using Real-Time PCR technique; B) western blotting bands; C) protein expression level of β-catenin. Bars in different letters represent significant difference.

A) gene expression level using Real-Time PCR technique; B) western blotting bands; C) protein expression level of α-SMA. Bars in different letters represent significant difference.

A) western blotting bands; B) protein expression level of ER. Bars in different letters represent significant difference.

A) western blotting bands; B) protein expression level of Ki-67. Bars in different letters represent significant difference.

Bars in different letters represent significant difference.

## Conclusion

In summary, we observed that the small molecule C-82 and naringenin could ameliorate inflammation, proliferation, and survival in menstrual blood-derived MenSCs from EM patients. Surprisingly, there was an increase in early apoptosis and a decrease in ROS, IL-6, IL-8 level, ER and Ki-67 protein expression, and α-SMA expression in E-MenSCs. Further investigations are needed to analyze the exact detailed effects of small molecule C-82 and naringenin, as herbal drugs, on endometriotic E-MenSCs. 

## Data Availability

Data will be made available upon request.

## Declaration

We have not used any AI tools or technologies to prepare this manuscript.
